# Floods and cause-specific mortality in the UK: a nested case-control study

**DOI:** 10.1186/s12916-024-03412-0

**Published:** 2024-05-07

**Authors:** Yao Wu, Danijela Gasevic, Bo Wen, Zhengyu Yang, Pei Yu, Guowei Zhou, Yan Zhang, Jiangning Song, Hong Liu, Shanshan Li, Yuming Guo

**Affiliations:** 1 School of Public Health and Preventive Medicine, Monash University, Level 2, 553 St Kilda Road, Melbourne, VIC 3004 Australia; 2 Department of Dermatology, Xiangya Hospital, Central South University, Changsha, 410008 Hunan China; 3 Department of Biochemistry and Molecular Biology, Monash Biomedicine Discovery Institute, Monash University, Melbourne, VIC 3800 Australia

**Keywords:** Mortality, Floods, Natural disaster, Long-term

## Abstract

**Background:**

Floods are the most frequent weather-related disaster, causing significant health impacts worldwide. Limited studies have examined the long-term consequences of flooding exposure.

**Methods:**

Flood data were retrieved from the Dartmouth Flood Observatory and linked with health data from 499,487 UK Biobank participants. To calculate the annual cumulative flooding exposure, we multiplied the duration and severity of each flood event and then summed these values for each year. We conducted a nested case-control analysis to evaluate the long-term effect of flooding exposure on all-cause and cause-specific mortality. Each case was matched with eight controls. Flooding exposure was modelled using a distributed lag non-linear model to capture its nonlinear and lagged effects.

**Results:**

The risk of all-cause mortality increased by 6.7% (odds ratio (OR): 1.067, 95% confidence interval (CI): 1.063–1.071) for every unit increase in flood index after confounders had been controlled for. The mortality risk from neurological and mental diseases was negligible in the current year, but strongest in the lag years 3 and 4. By contrast, the risk of mortality from suicide was the strongest in the current year (OR: 1.018, 95% CI: 1.008–1.028), and attenuated to lag year 5. Participants with higher levels of education and household income had a higher estimated risk of death from most causes whereas the risk of suicide-related mortality was higher among participants who were obese, had lower household income, engaged in less physical activity, were non-moderate alcohol consumers, and those living in more deprived areas.

**Conclusions:**

Long-term exposure to floods is associated with an increased risk of mortality. The health consequences of flooding exposure would vary across different periods after the event, with different profiles of vulnerable populations identified for different causes of death. These findings contribute to a better understanding of the long-term impacts of flooding exposure.

**Supplementary Information:**

The online version contains supplementary material available at 10.1186/s12916-024-03412-0.

## Background

Floods are the most frequent type of weather-related disaster, accounting for about 47% of all weather-related disasters from 1995 to 2015 [1, 2]. Between 1995 and 2015, more than 2.3 billion people were affected by flood disasters, with over 157 thousand people dying directly as a result of floods [3]. In recent years, many intense urban flooding events have been recorded in the UK, resulting in loss of lives, damages to personal property and public health infrastructure, and disruption to vital services such as water, communications, energy, and public transport [4–9]. Approximately 1.9 million people across the UK are at risk of floods, and this number will double as early as the 2050s [10].

In addition to immediate fatalities due to drowning and acute trauma [11], floods can also cause short- (lasting days or weeks) or medium-health impacts (several weeks or months), including the spread of water- and vector-borne diseases, such as cholera, typhoid, or malaria; injuries during evacuations and disaster clean-up; and exposure to chemical hazards [1, 12]. Non-communicable diseases (e.g. cardiovascular disease, neoplasms, chronic respiratory diseases, and diabetes) which need prolonged treatment and care can be exacerbated after floods due to a disruption in care, treatment, medication, supplies, equipment, and overcrowding in shelters [13–18]. Mental health issues may arise from stressors caused by floods (e.g. property damage, financial loss, loss of a loved one) and have long-lasting health effects on mortality and morbidity. These long-term health consequences may arise from several pathways, including impairment of the immune system, sleep disturbances, substance abuse, and inadequate self-care [19–22].

Despite the severe impacts of floods, there currently is limited epidemiological evidence on the long-term mortality impacts of exposure to floods. To address these gaps in knowledge, we utilized the UK Biobank project, a population-based study with a large sample size, to explore the long-term effects of flooding on mortality. We aimed to estimate the risk of all-cause and seven cause-specific mortality associated with floods and to explore the lag patterns in mortality risk. We also conducted subgroup analyses to identify populations who are potentially more vulnerable to flood-related death.

## Methods

### Study design and study population

**Fig. 1:**
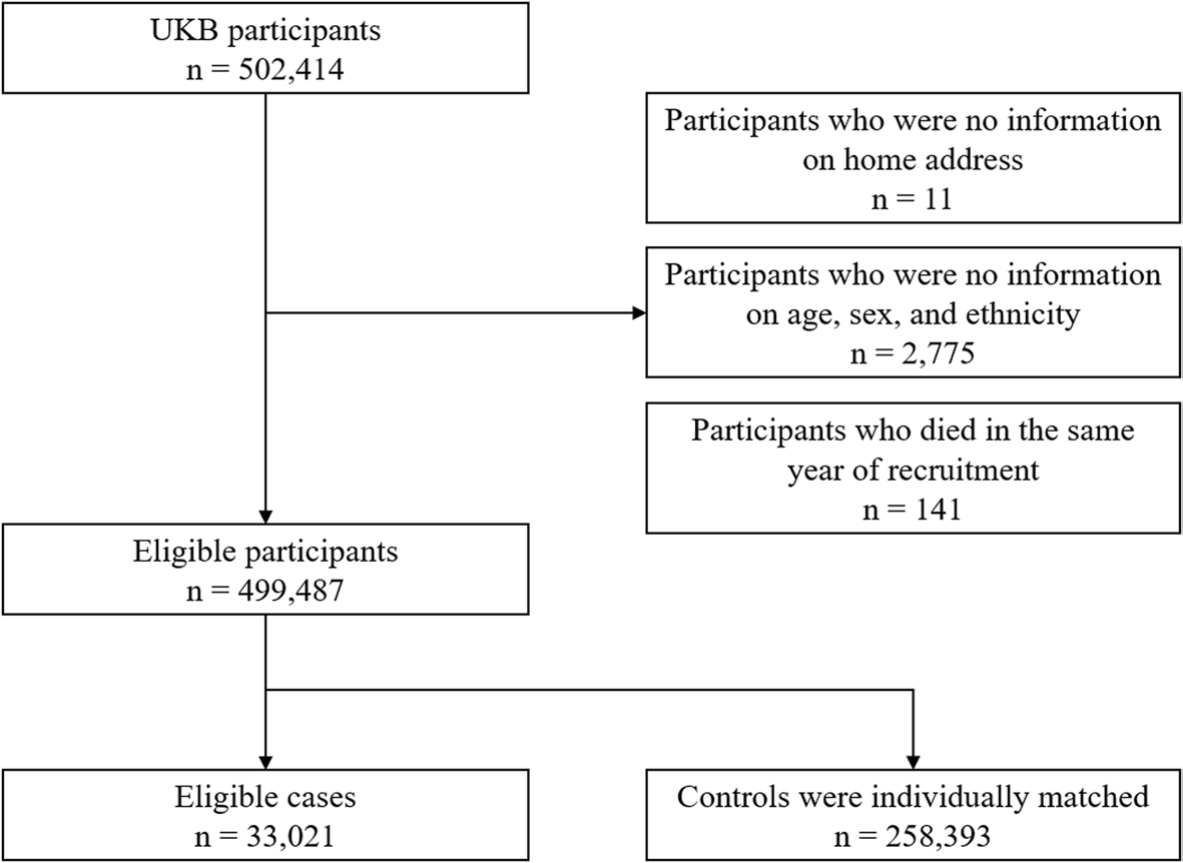
A flow diagram to show participants whose data were used to estimate the association between flooding exposure and mortality

We conducted a nested case-control study within a cohort of participants registered with the UK Biobank study. About 0.5 million residents aged between 37 and 73 years were enrolled in the UK Biobank from 2006 to 2010, from 21 assessment centres across England, Wales, and Scotland. The cohort was followed up until the date of death or the study end date (December 31, 2020). We excluded participants lacking longitude and latitude data of residence (*n* = 11), participants with missing data on age, sex, and ethnicity (*n* = 2775), and those who died in the year of recruitment (*n* = 141). A total of 499,487 participants were included (Fig 1). All participants in the UK Biobank study provided informed consent. The utilization of the data presented in this paper has been approved by the UK Biobank access committee under UK Biobank application number 55257.

### Case-control selection

With the nested case-control design, we matched controls to cases with replacement at the time of the outcome event and assessed exposure retrospectively, from the date of death or end of follow-up. This ensures identical exposure lengths across participants. Using a risk-set sampling method, each case was matched with eight controls randomly selected from study participants who met the matching criteria for age (within 5 years), sex (male and female), and ethnicity (White, Black, Asian or Asian British, mixed, Chinese, and others). The index date for cases corresponded to the date of death; while for controls, it was the date of death of the matched case participant. For twelve case-control sets, eligible controls were less than eight but at least one (Fig 1).

Participants were eligible for inclusion as cases for the study if they died during the study period. We defined all-cause mortality and seven cause-specific mortality categories using the International Classification of Diseases, edition 10 (ICD-10), classification as follows: neoplasms, C00–D48; cardiovascular disease, I00–I99; respiratory diseases, J09–J98; digestive disease, K20–K93; neurodegenerative disease, F01–03, G122, G20, G21, G23, G30, G31; mental and behavioural disorders: F00–F90; and suicide: X60–X84, Y10–Y34, Y87.

### Flooding exposure

We collected flood data during 2000–2020 from the Dartmouth Flood Observatory (DFO), which is a global catalogue of all flood events with detailed information on start date, end date, centroids, impacted geographic areas, and severities. All documented flood events were sourced from news, government, and instrumental sources and have been validated by satellite observations [23, 24]. Participants whose home addresses fall within flood-affected areas were considered as having been exposed to a flood event. To assess the long-term effect of floods, we calculated a cumulative exposure during the study period for each participant. Building on previous research [25, 26], we derived the annual cumulative exposure by multiplying the duration and severity of each flood event and summing these values for each year. Our preliminary analyses suggested a weak negative association between flood severity and duration (Pearson coefficient: − 0.03). The severity of each flood event documented in the DFO was classified based on a pre-defined scale, detailed in Additional file 1: Table S1. For each participant, annual cumulative flooding exposure was calculated using equation (1):

1 $$\mathrm{Flood}\;{\mathrm{index}}_{i,\text{year}=m}=\sum_{j=1}^n{\text{Duration}}_{ij}\times{\text{Severity}}_{ij}(j=1,\dots,n)$$

where $\mathrm{Flood}\;{\mathrm{index}}_{i,\text{year}=m}$ stands for the cumulative flooding exposure in year $m$ for participants $i$. ${{\text{Duration}}}_{ij}$ and ${{\text{Severity}}}_{ij}$ represent the duration (day) and the severity of the $j$ th flood event that participant $i$ experienced in year $m$, respectively. If there were no flood events within a given year, a flood index of 0 was recorded.

### Meteorological data

We extracted hourly temperature and relative humidity data from the European Centre for Medium-Range Weather Forecasts Reanalysis v5 (ERA-5) reanalysis data set with a spatial resolution of 0.1°×0.1°. We mapped meteorological data to the participant’s geocoded residential address at baseline. Daily meteorological data were calculated by averaging hourly data within each day. Daily temperature and relative humidity were then aggregated into yearly averages.

### Covariates

Baseline data collected by the UK Biobank include demographics, lifestyle factors, socioeconomic status, and anthropometric measurements. We included additional covariates informed by existing literature, beyond those used for matching cases and controls [27–29]: body mass index (BMI), physical activity, healthy diet score, cigarette smoking, alcohol consumption, educational attainment, average total annual household income before tax, Townsend deprivation index (TDI), overall health rating, and assessment centres. BMI was calculated from objectively measured weight and height as weight over height squared and expressed as kg/m^2^. Physical activity was derived from the International Physical Activity Questionnaire-Short Form (IPAQ-SF) [30]. Participants were categorized at ‘high’ (≥ 1500 metabolic equivalent (MET)-minutes/week), ‘moderate’ (≥ 600 MET-minutes/week), or ‘low’ levels of physical activity following standardized IPAQ-SF scoring guidance [30]. Diet score was calculated based on the following dietary factors: vegetable intake ≥ 3 servings/day; fruit intake ≥ 3 servings /day; whole grains ≥ 3 servings/day; refined grains ≤ 1.5 servings/day; fish intake ≥ 2 servings/day; unprocessed red meat intake ≤ 2 servings/week; and processed meat intake ≤ 2 servings/week. Each point was given for each favourable dietary factor, and the suboptimal diet was defined as a diet score < 4. Smoking status was coded into three categories: current, former, and never. Low-risk alcohol consumption was defined as moderate drinking (no more than one drink/day for women and two drinks/day for men; one drink is measured as 8 g ethanol in the UK) on a relatively regular frequency [31]. Educational attainment was coded in two categories: ‘high’ (college or university degree) or ‘low’ (A/AS levels or equivalent, O levels/GCSEs or equivalent, or none of the above). Annual household income was classified into two groups (< £31,000 and ≥ £31,000). TDI was utilized to define area deprivation level, with participants being classified as either high (TDI above the median) or low [32]. Self-reported health was categorized as poor, fair, good, and excellent [27].

### Statistical analysis

We performed conditional logistic regression analysis to estimate the risk of mortality associated with per unit increase in flood index. Year-specific flood index was modelled using a distributed lag non-linear model featuring a non-linear exposure-response association and the additional lag-response association, respectively [33–36]. The lag-response association refers to how the risk changes over time and provides an estimation of the combined immediate and delayed effects that accumulate throughout the lag period. We first modelled the exposure-response curve with a natural cubic spline with three degrees of freedom. However, the nonlinear analysis indicated an approximately linear relationship (Additional file 1: Fig. S1). Further, both the Akaike Information Criterion (AIC) and the Bayesian Information Criterion (BIC) favoured the linear model (Additional file 1: Table S2). Therefore, we applied a linear exposure-response relationship in the formal analysis. The lag-response curve was modelled with a natural cubic spline with three degrees of freedom plus an intercept. The exposure window comprised the 0 to 5 years before the index date. A maximum lag of 5 years was used because the flood-related mortality risk declined to zero by the lag year 5.

Estimates of risk were obtained from the crude model that only included flood (model 1); the multivariate model that additionally controlled for socioeconomic status (education attainment, household income, and deprivation) (model 2); and the full model that additionally adjusted for BMI, physical activity, smoking, alcohol consumption, suboptimal diet, overall health rating, mean temperature, mean relative humidity, and assessment centre which serves as an indicator of the recruitment location for each participant (model 3). All variance inflator factors were less than 1.5, indicating no multicollinearity. Temperature and relative humidity terms were defined as the average annual mean temperature and relative humidity over 6 years (lag 0–5 years) preceding the index date, respectively. Given that the crude model (model 1) did not include any covariates, all participants were retained in the analysis. For models 2 and 3, we excluded participants with any missing data. In sensitivity analyses, we employed multiple imputation to address missing covariate data and assess the robustness of our findings.

We further identified subgroups vulnerable to floods through stratification analyses by age group (≤64 and >65 years), sex, weight status defined according to BMI (≤ 24.9, 25–29.9, ≥ 30), education attainment, household income, physical activity, suboptimal diet, alcohol consumption status, smoking status, and area deprivation level. Results are presented as odds ratios (ORs) and their 95% confidence intervals (95% CIs) per unit increase in flood index. The significance of the difference in results between subgroups was tested using a random-effect meta-regression model.

### Sensitivity analysis

We carried out the following sensitivity analyses: (1) Multiple imputation by chained equations was used for the missing values. Five imputed data sets were created, and their results were combined using Rubin’s rules [37]. (2) Alternative degrees of freedom were used for the lag-response association of flood. (3) Alternative degrees of freedom were used for the non-linear exposure-response relationship of mean temperature and relative humidity. (4) Alternative matching ratios (1:4 and 1:6) were used. (5) Excluding data after 2020 to control for the effect of the COVID-19 pandemic. (6) To capture the variation in flooding impacts within the year preceding mortality, we performed additional analyses with monthly flood index.

## Results

Table 1 shows the baseline characteristics of the 33,021 death cases and the 258,393 matched controls. The mean age (± standard deviation (SD)) of participants at study entry was 61.3 (± 6.4) years; 170,549 (58.5%) were male; 281,175 (96.5%) were white. Participants who died were more likely to have a higher BMI and lower household income; were less likely to be university graduates; more likely to smoke; and consumed less fruit and vegetables and more red and processed meat. They were also more likely to rate their overall health as poor and fair. Baseline characteristics of cases and controls with any missing values in covariates are shown in Additional file 1: Table S3.

**Table 1:** Baseline characteristics of cases and matched controls enrolled in UK Biobank

	**Overall**	**Case**	**Control**
*N*	291,414	33,021	258,393
Age, mean (SD)	61.3 (6.4)	61.6 (6.5)	61.3 (6.4)
Male, *n* (%)	170,549 (58.5)	19,591 (59.3)	150,958 (58.4)
White ethnicity, *n* (%)	281,175 (96.5)	31,867 (96.5)	249,308 (96.5)
BMI, mean (SD)	27.6 (4.6)	28.3 (5.4)	27.6 (4.5)
High education attainment, *n* (%)	83,107 (29.1)	7528 (23.4)	75,579 (29.8)
Household income ≥31000, *n* (%)	101,733 (41.9)	8200 (30.7)	93,533 (43.3)
Physical activity, *n* (%)			
Low	42,663 (18.4)	6104 (24.1)	36,559 (17.6)
Middle	57,267 (24.6)	6223 (24.6)	51,044 (24.6)
High	132,489 (57.0)	12,956 (51.2)	119,533 (57.7)
Smoking, *n* (%)			
Never	144,356 (49.8)	12,588 (38.4)	131,768 (51.2)
Previous	116,627 (40.2)	13,790 (42.0)	102,837 (40.0)
Current	29,076 (10.0)	6417 (19.6)	22,659 (8.8)
Non-moderate alcohol consumer, *n* (%)	164,134 (75.6)	17,408 (77.9)	146,726 (75.3)
Suboptimal diet, *n* (%)	122,724 (43.6)	15,160 (48.3)	107,564 (43.0)
High Townsend deprivation index, *n* (%)	139,808 (48.0)	18,529 (56.2)	121,279 (47.0)
Health rating, *n* (%)			
Poor	14,296 (4.9)	4383 (13.4)	9913 (3.9)
Fair	64,233 (22.1)	10,066 (30.8)	54,167 (21.0)
Good	167,285 (57.7)	15,241 (46.6)	152,044 (59.1)
Excellent	44,269 (15.3)	3025 (9.2)	41,244 (16.0)

*N* number, *SD* standard deviation

The distributions of the flood index and meteorological factors are shown in Table 2. The annual average flood index across all participants during the study period ranged from 0.0 to 38.3, with a median value of 1.8 (25^th^ to 75^th^ percentiles: 0.5 to 3.6). Cases exposed to higher levels of flooding than controls during the 6 years before the end of follow-up (Additional file 1: Fig. S2). The median annual mean temperature was 10.0 °C (25^th^ to 75^th^ percentiles: 9.3°C to 10.7°C) (Table 2). The flood index was negatively correlated with mean temperature (Pearson *r* = − 0.04) but positively correlated with relative humidity (Pearson *r* = 0.08).

**Table 2:** Distribution of annual average flood index and meteorological factors

**Variables**	**Mean**	**SD**	**Minimum**	**P**_**25**_	**Median**	**P**_**75**_	**Maximum**
Flood index	4.4	6.4	0.0	0.5	1.8	3.6	38.3
Mean temperature (°C)	9.9	0.8	5.9	9.3	10.0	10.7	12.0
Relative humidity (%)	80.4	1.5	77.2	79.3	80.2	81.4	87.2

*Abbreviation*: *SD*, standard deviation; *P*_25_, the 25th percentile; *P*_75_, the 75th percentile

**Fig. 2:**
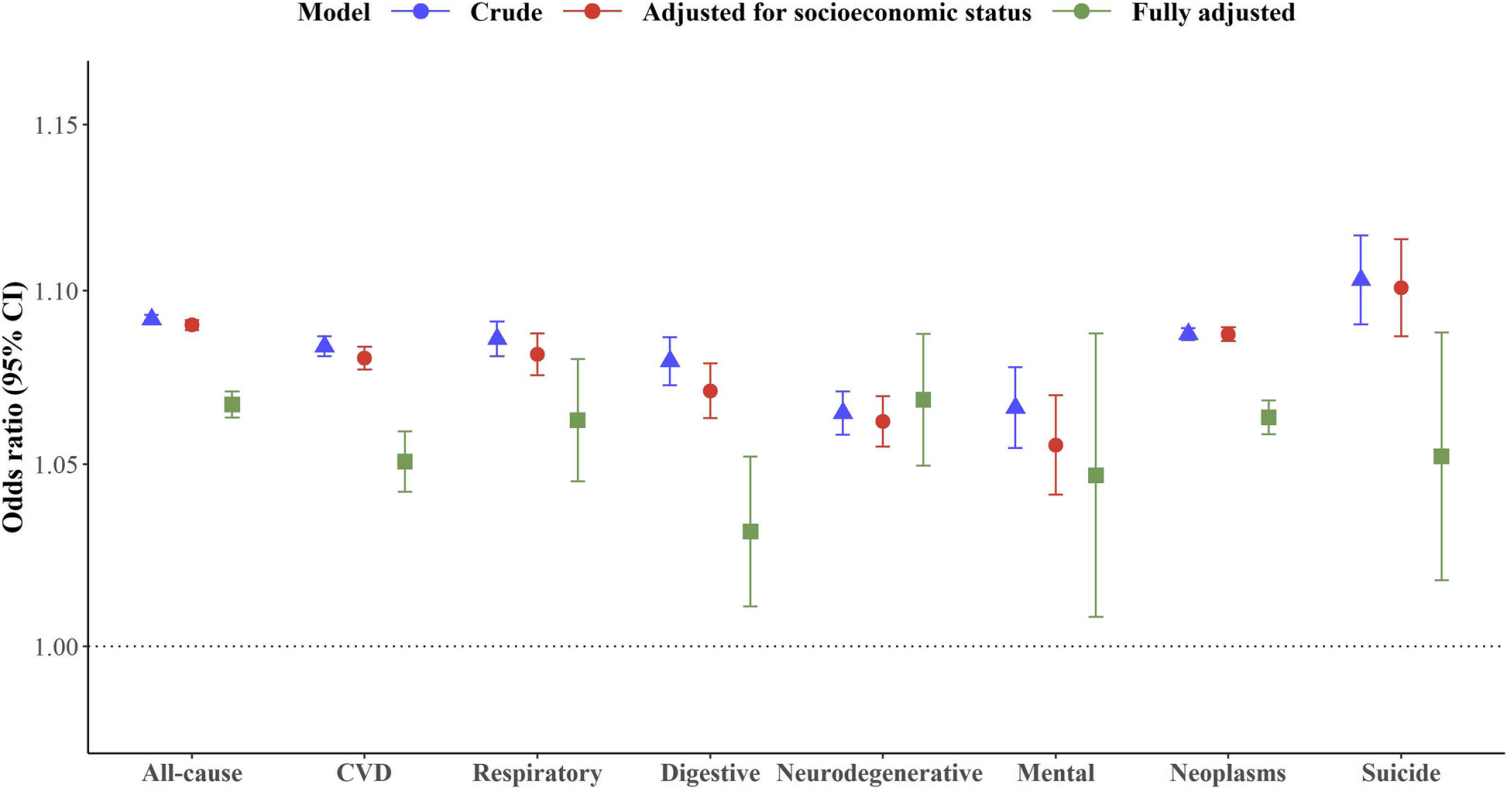
Cumulative odds ratio of all-cause and cause-specific mortality associated with per unit increase in flood index over lag years 0–5. Estimates of risk were obtained from the crude model that only included flood (crude); the multivariate model that additionally controlled for socioeconomic status (education attainment, household income, and deprivation); and the full model that additionally adjusted for BMI, physical activity, smoking, alcohol consumption, suboptimal diet, overall health rating, mean temperature, mean relative humidity, and assessment centre which serves as an indicator of the recruitment location for each participant (fully adjusted). The error bars represent 95% confidence intervals

Figure 2 illustrates the estimated cumulative OR of all-cause and cause-specific mortality associated with per unit increase in flood index over lag years 0–5. Per unit increase in flood index was associated with a 9.2% increased risk of all-cause mortality (OR: 1.092, 95% CI: 1.090–1.093) in the crude model. The results remained similar after further adjustment for socio-economic status (OR: 1.090, 95% CI: 1.088–1.091), whereas adjustment for lifestyle factors decreased the strength of the association (OR for fully adjusted model: 1.067, 95% CI: 1.063–1.071). Similar effects were observed for cause-specific mortality after fully adjusting the models, whereby a greater flood index was associated with a greater risk of death from neurodegenerative diseases (OR: 1.068, 95% CI: 1.050–1.087), neoplasm (OR: 1.063, 95% CI: 1.058–1.068), respiratory diseases (OR: 1.062, 95% CI: 1.045–1.080), suicide (OR: 1.052, 95% CI: 1.018–1.088), cardiovascular diseases (OR: 1.051, 95% CI: 1.042–1.059), mental diseases (OR: 1.047, 95% CI: 1.008–1.087), and digestive diseases (OR: 1.031, 95% CI: 1.011–1.052) (Fig. 2, Additional file 1: Table S4).

**Fig. 3:**
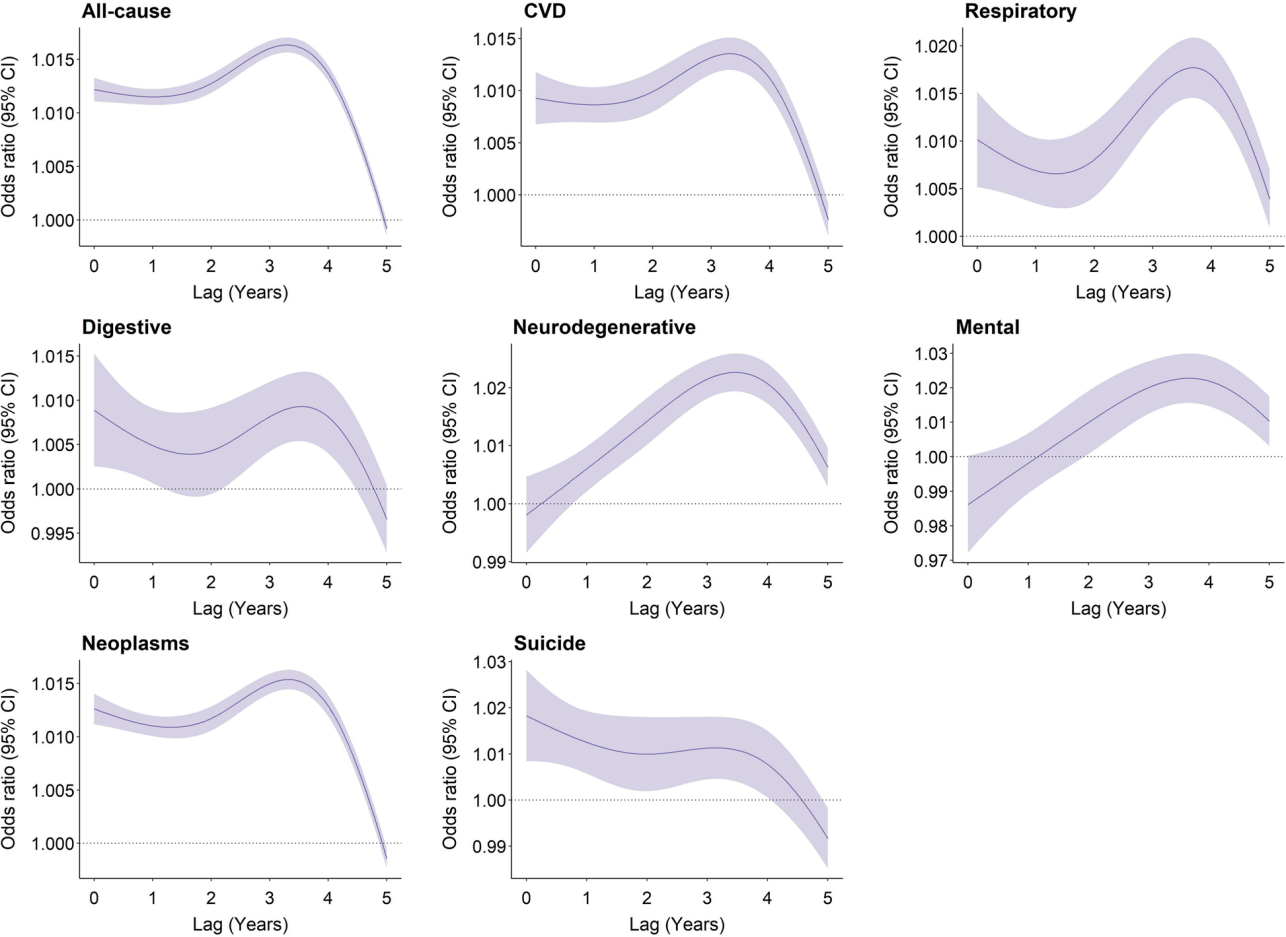
Overall lag structure in effects of flooding exposure on cause-specific mortality. Shaded areas represent 95% confidence intervals for the odds ratio

Figure 3 shows the lag structure in the effects of flooding exposure on all-cause and cause-specific mortality. For all-cause mortality, the magnitude of associations increased from the current year (OR: 1.012, 95% CI: 1.011–1.013) to the lag year 3 (OR: 1.016, 95% CI: 1.015–1.017), and subsequently diminished to zero by lag year 5. For neurodegenerative mortality and mortality due to mental-ill health, the mortality risk was negligible in the current year, but strongest in the lag years 3 and 4. By contrast, the risk of mortality from suicide was the strongest in the current year (OR: 1.018, 95% CI: 1.008–1.028), and attenuated to lag year 5.

Subgroup analyses revealed that participants with higher levels of education and household income had a higher estimated risk of death from most causes in association with flooding exposure. Participants aged below 64 and female had a higher estimated risk of death from all-cause mortality, respiratory diseases, and neoplasm, but a lower estimated risk of death from digestive and mental diseases, respectively. The risk of suicide-related mortality in association with flooding exposure was higher among participants who were obese, had lower household income, engaged in less physical activity, were non-moderate alcohol consumers, and had high deprivation levels (Table 3).

**Table 3:** Subgroup analyses for the associations of flooding exposure with all-cause and cause-specific mortality

**Subgroup**	**All-cause**		**CVD**		**Respiratory**		**Digestive**		**Neurodegenerative**		**Mental**		**Neoplasm**		**Suicide**	
	**Odds ratio (95% CI)**	*p*-value *	**Odds ratio (95% CI)**	***p*-value**	**Odds ratio (95% CI)**	***p*-value**	**Odds ratio (95% CI)**	***p*-value**	**Odds ratio (95% CI)**	***p*-value**	**Odds ratio (95% CI)**	***p*-value**	**Odds ratio (95% CI)**	***p*-value**	**Odds ratio (95% CI)**	***p*-value**
Age																
≤ 64 years	1.072 (1.067 to 1.077)	Ref	1.055 (1.044 to 1.066)	Ref	1.086 (1.060 to 1.112)	Ref	1.019 (0.995 to 1.043)	Ref	1.064 (1.036 to 1.092)	Ref	1.035 (0.954 to 1.123)	Ref	1.069 (1.063 to 1.075)	Ref	1.054 (1.018 to 1.092)	Ref
> 65 years	1.057 (1.051 to 1.064)	< 0.001	1.046 (1.033 to 1.060)	0.329	1.043 (1.020 to 1.068)	0.019	1.082 (1.041 to 1.124)	0.01	1.074 (1.047 to 1.101)	0.6	1.044 (0.998 to 1.091)	0.862	1.052 (1.044 to 1.060)	< 0.001	1.113 (0.826 to 1.500)	0.723
Sex																
Female	1.079 (1.073 to 1.086)	Ref	1.055 (1.033 to 1.077)	Ref	1.093 (1.059 to 1.128)	Ref	1.005 (0.960 to 1.052)	Ref	1.087 (1.054 to 1.121)	Ref	0.933 (0.831 to 1.047)	Ref	1.075 (1.067 to 1.083)	Ref	1.054 (0.969 to 1.147)	Ref
Male	1.060 (1.055 to 1.064)	< 0.001	1.050 (1.041 to 1.060)	0.732	1.049 (1.028 to 1.069)	0.028	1.035 (1.012 to 1.059)	0.252	1.058 (1.035 to 1.082)	0.156	1.060 (1.017 to 1.106)	0.04	1.056 (1.050 to 1.062)	< 0.001	1.050 (1.009 to 1.093)	0.932
BMI																
≤ 24.9	1.072 (1.064 to 1.079)	Ref	1.059 (1.039 to 1.079)	Ref	1.074 (1.042 to 1.107)	Ref	1.063 (1.020 to 1.108)	Ref	1.077 (1.046 to 1.109)	Ref	1.027 (0.960 to 1.098)	Ref	1.066 (1.057 to 1.075)	Ref	1.069 (0.986 to 1.160)	Ref
25~29.9	1.067 (1.062 to 1.073)	0.357	1.052 (1.039 to 1.065)	0.555	1.059 (1.031 to 1.088)	0.499	1.020 (0.985 to 1.057)	0.136	1.072 (1.043 to 1.103)	0.833	1.028 (0.968 to 1.091)	0.986	1.060 (1.053 to 1.068)	0.371	1.119 (1.051 to 1.192)	0.384
≥ 30	1.059 (1.051 to 1.066)	0.012	1.045 (1.031 to 1.059)	0.279	1.057 (1.025 to 1.090)	0.474	1.027 (0.993 to 1.063)	0.208	1.033 (0.991 to 1.077)	0.106	1.099 (1.003 to 1.204)	0.243	1.058 (1.049 to 1.068)	0.274	7.396 (6.789 to 8.058)	<0.001
Education																
Low	1.055 (1.051 to 1.060)	Ref	1.040 (1.030 to 1.051)	Ref	1.059 (1.039 to 1.079)	Ref	1.029 (1.004 to 1.054)	Ref	1.058 (1.035 to 1.082)	Ref	1.048 (1.003 to 1.095)	Ref	1.051 (1.045 to 1.057)	Ref	1.078 (1.027 to 1.133)	Ref
High	1.085 (1.078 to 1.091)	< 0.001	1.075 (1.059 to 1.092)	<0.001	1.076 (1.041 to 1.113)	0.405	1.040 (0.998 to 1.084)	0.637	1.079 (1.043 to 1.116)	0.355	1.051 (0.963 to 1.146)	0.961	1.080 (1.072 to 1.089)	< 0.001	1.039 (0.967 to 1.117)	0.407
Household income																
≤ 31,000	1.052 (1.047 to 1.057)	Ref	1.039 (1.029 to 1.050)	Ref	1.054 (1.034 to 1.074)	Ref	1.020 (0.996 to 1.044)	Ref	1.069 (1.046 to 1.093)	Ref	1.067 (1.023 to 1.114)	Ref	1.051 (1.044 to 1.057)	Ref	1.109 (1.044 to 1.177)	Ref
> 31,000	1.087 (1.081 to 1.093)	< 0.001	1.076 (1.060 to 1.092)	<0.001	1.092 (1.054 to 1.131)	0.08	1.074 (1.028 to 1.121)	0.039	1.060 (1.026 to 1.095)	0.671	0.978 (0.880 to 1.087)	0.133	1.078 (1.071 to 1.086)	< 0.001	1.015 (0.968 to 1.065)	0.024
Alcohol consumption																
Non-moderate	1.070 (1.062 to 1.078)	Ref	1.045 (1.028 to 1.063)	Ref	1.046 (1.009 to 1.085)	Ref	1.066 (1.010 to 1.125)	Ref	1.067 (1.031 to 1.103)	Ref	1.044 (0.960 to 1.134)	Ref	1.061 (1.050 to 1.072)	Ref	1.287 (1.212 to 1.367)	Ref
Moderate	1.066 (1.062 to 1.070)	0.442	1.052 (1.043 to 1.062)	0.498	1.067 (1.047 to 1.087)	0.349	1.023 (0.999 to 1.046)	0.167	1.071 (1.048 to 1.094)	0.848	1.039 (0.994 to 1.087)	0.932	1.063 (1.058 to 1.069)	0.689	1.048 (1.010 to 1.087)	<0.001
Smoking																
Never	1.082 (1.076 to 1.089)	Ref	1.064 (1.048 to 1.080)	Ref	1.067 (1.029 to 1.106)	Ref	1.044 (1.002 to 1.087)	Ref	1.075 (1.045 to 1.104)	Ref	1.061 (0.990 to 1.136)	Ref	1.077 (1.070 to 1.085)	Ref	1.045 (0.988 to 1.105)	Ref
Previous	1.061 (1.055 to 1.067)	< 0.001	1.049 (1.038 to 1.061)	0.157	1.063 (1.038 to 1.088)	0.864	1.049 (1.018 to 1.082)	0.834	1.048 (1.019 to 1.077)	0.204	1.006 (0.946 to 1.070)	0.265	1.056 (1.049 to 1.064)	< 0.001	1.119 (1.040 to 1.205)	0.145
Current	1.048 (1.039 to 1.056)	< 0.001	1.033 (1.014 to 1.053)	0.018	1.049 (1.016 to 1.084)	0.511	1.011 (0.965 to 1.059)	0.314	1.104 (1.034 to 1.179)	0.45	1.213 (1.036 to 1.421)	0.126	1.041 (1.029 to 1.053)	< 0.001	1.101 (0.957 to 1.267)	0.496
Physical activity																
Low	1.054 (1.046 to 1.062)	Ref	1.047 (1.029 to 1.066)	Ref	1.065 (1.030 to 1.102)	Ref	1.010 (0.972 to 1.049)	Ref	1.057 (1.014 to 1.100)	Ref	1.074 (0.977 to 1.180)	Ref	1.054 (1.042 to 1.065)	Ref	1.349 (1.259 to 1.447)	Ref
Middle	1.068 (1.061 to 1.075)	0.014	1.039 (1.021 to 1.057)	0.528	1.045 (1.011 to 1.079)	0.415	1.046 (1.004 to 1.090)	0.215	1.065 (1.030 to 1.101)	0.773	1.019 (0.943 to 1.100)	0.397	1.057 (1.047 to 1.067)	0.659	1.166 (1.004 to 1.354)	0.083
High	1.071 (1.066 to 1.076)	< 0.001	1.056 (1.044 to 1.067)	0.446	1.073 (1.046 to 1.100)	0.739	1.045 (1.012 to 1.079)	0.171	1.071 (1.043 to 1.100)	0.581	1.041 (0.981 to 1.104)	0.585	1.068 (1.061 to 1.074)	0.031	1.067 (1.018 to 1.117)	<0.001
Suboptimal diet																
No	1.069 (1.064 to 1.075)	Ref	1.054 (1.042 to 1.067)	Ref	1.084 (1.058 to 1.110)	Ref	1.049 (1.017 to 1.082)	Ref	1.058 (1.033 to 1.083)	Ref	1.045 (0.993 to 1.100)	Ref	1.066 (1.060 to 1.073)	Ref	1.077 (1.022 to 1.134)	Ref
Yes	1.064 (1.058 to 1.069)	0.14	1.048 (1.036 to 1.060)	0.468	1.045 (1.022 to 1.069)	0.031	1.024 (0.996 to 1.054)	0.276	1.082 (1.051 to 1.113)	0.24	1.049 (0.983 to 1.120)	0.921	1.060 (1.052 to 1.067)	0.179	1.037 (0.978 to 1.100)	0.349
Deprivation																
Low	1.071 (1.065 to 1.076)	Ref	1.059 (1.046 to 1.072)	Ref	1.058 (1.030 to 1.086)	Ref	1.055 (1.021 to 1.091)	Ref	1.064 (1.038 to 1.090)	Ref	1.029 (0.973 to 1.088)	Ref	1.061 (1.054 to 1.068)	Ref	0.987 (0.929 to 1.049)	Ref
High	1.061 (1.056 to 1.067)	0.019	1.042 (1.031 to 1.053)	0.054	1.066 (1.043 to 1.089)	0.667	1.018 (0.992 to 1.045)	0.096	1.070 (1.042 to 1.099)	0.755	1.068 (1.013 to 1.126)	0.348	1.062 (1.055 to 1.069)	0.783	1.089 (1.037 to 1.144)	0.013

Data are odds ratios per unit increase in flood index. The model was adjusted for matching factors, socio-economic factors, BMI, physical activity, smoking, alcohol consumption, diet score, overall health rating, and assessment centre

^*^p-value for the difference

Our sensitivity analysis suggested that using multiple imputed data did not change study findings (Additional file 1: Fig. S3). Our results were not dependent on modelling assumptions and remained unaffected by the COVID-19 pandemic (Additional file 1: Fig. S4–7). Matching ratios of 1:4 and 1:6 revealed a modest increase in the odds ratio for all-cause mortality and neoplasms, while the odds ratio for other causes of death remained unchanged **(**Additional file 1: Fig. S8**).** For all-cause mortality, with per unit increase in monthly flood index, odds ratios over 0–12 months preceding the mortality ranged from 1.005 (95% CI: 1.005–1.006) to 1.018 (95% CI: 1.017–1.018) (Additional file 1: Table S5).

## Discussion

In this nested case-control study, we observed a significantly increasing risk of mortality associated with floods. The exposure-response curve was linear, with no discernible thresholds. The lag pattern varied across different causes of death. Flooding exposure has a long-lasting impact on neurodegenerative and mental diseases, whereas it has an immediate impact on suicide. Subgroup analyses revealed specific groups of vulnerable populations for flood-related death, which varied according to the cause of death.

Every unit increase in flood index was associated with a 6.7% increase in all-cause mortality risk over the following 6 years. This finding was similar to cause-specific mortality. Although few epidemiological studies have assessed the long-term effect of floods on mortality, our findings are consistent with previous findings of short-term flooding exposure showing increased risk of cholera at lag 0–20 weeks [38], diarrhoea at lag 0–28 weeks [38], respiratory infection at lag 3 months [39], typhoid fever at lag 1 week [40], malaria at lag 1 year [12], malnutrition at lag 1 year [41], and mental disorders at lag 6 months [19]. One study assessing the effects of flooding on mortality in England and Wales during 1994–2005 suggested a deficit of deaths in the post-flood period [42]. The inconsistency might result from the underestimation of death number, which can occur when deaths are registered at different places after displacement and a short observation period (one year after flooding exposure) during which the occurrence of death has not been observed. Milojevic et al. reported a slight but non-significant increase in mortality rates following the floods in Bangladesh in the flooded areas compared to non-flooded areas [43]. The accuracy of their results might be subject to recall bias in exposure assessment, given that exposure to flooding was ascertained from an interview survey four years after the flood event.

The long-term health deterioration resulting from floods could be attributed to mental health disorders driven by financial losses and community or social disruption, especially for those who live in resource-poor countries and communities (e.g. floodplains or non-resistant buildings, lack of warning systems and awareness of flooding hazard) [1, 44]. For example, previous studies reported a significant and continued increase in the prevalence of post-traumatic stress disorder (PTSD), stress, anxiety, depression, and even suicide ideation following flooding exposure [20], which contribute to worse health outcomes. Additionally, among people who were exposed to floods, those who had chronic medical conditions are at higher risk of health deterioration due to potential disruptions in medication and healthcare services. Previous studies have noted that older adults and those receiving long-term care services showed decreased treatment adherence (e.g. interruption of medication and access to physicians) months and even years after the flooding [45, 46], which serve to exacerbate or prolong symptoms of existing conditions.

Flooding exposure has a long-lasting impact on neurodegenerative and mental diseases, reaching its peak at 3–4 years post-exposure whereas the highest risk of mortality due to suicide occurs in the year of exposure. This suggests that varying health issues should be given consideration, depending on the stage following flooding exposure. A study in Queensland has noted that direct exposure to flood resulted in an increase in alcohol and tobacco usage half a year after flooding [46] and use of substances has been associated with an increased risk of suicide attempts in previous studies [47–50]. However, social support and compensation coverage have been demonstrated to have had a positive impact on health [20], which helps reduce the risk of subsequent suicide attempts. By contrast, cognitive decline is more likely to occur 2 years later after natural disasters [51, 52], resulting from the new onset of depression and disruption of social contacts (e.g. loss of interactions with neighbours) [51].

Our findings align with previous studies highlighting the vulnerability of cancer patients to disruptions in healthcare services following natural disasters. While limited evidence suggests an increased risk of disease exacerbation among cancer patients post-disaster, our primary concern is the potential for delays in receiving essential cancer care [14, 18]. Natural disasters like floods can severely disrupt healthcare systems, leading to damage to oncology centres, loss of medical records, pharmaceutical shortages, displacement of healthcare workers, and disruptions in pathology specimen handling, all of which can compromise cancer patient care [13, 53–56]. The relocation of cancer patients to temporary shelters can be particularly challenging and distressing, especially for those with clinical instability [53]. Additionally, initial recovery efforts following natural disasters often prioritize immediate needs such as providing shelter, food, water, and addressing injuries from environmental hazards, infectious diseases, or other acute conditions [57]. This prioritization of immediate needs may inadvertently overlook the continuity of care required for non-acute medical issues like cancer. Given the individualized and continuous nature of cancer treatment, neoplasms are particularly susceptible to the disruptions caused by natural disasters. Our study demonstrates the association between floods and elevated mortality risks among cancer patients, reinforcing the urgent need to prioritize the needs of cancer patients before, during, and after disasters [13, 58].

Profiles of vulnerable populations to flood-related mortality varied across causes of death. Of all factors considered, socio-economic status, which is determined by individual levels of education and income, has been identified as a significant modifier of flood-related mortality impacts. Individuals with higher socioeconomic status tend to have an increased risk of flood-related mortality from chronic diseases (e.g. cardiovascular diseases, respiratory diseases, and neurodegenerative diseases) but decreased risk of flood-related mortality from suicide. Although there is very limited evidence that can elucidate this finding, some insights can be gathered from the following studies. It is reported that people in high socioeconomic groups are more likely to be affected by work-life conflict-induced mental illness due to their higher occupational aspirations but a greater discrepancy between aspirations and reality [59, 60]. Flooding exposure may further amplify the disparity between an individual’s aspirations and their actual circumstances, resulting in a negative impact on their mental health. Long-lasting psychological illness has been associated with worse chronic medical conditions [61].

In our study, we observed that participants with higher BMI and lower physical activity levels exhibited a significantly higher risk of flood-related mortality from suicide, but a comparatively lower risk of all-cause mortality. These associations can be attributed to different factors. On the one hand, individuals in low socio-economic groups, those engaged in minimal physical activity, and non-moderate alcohol drinkers, are at higher risk of developing suicide ideation in a short time following psychological trauma associated with flooding exposure [49, 62]. On the other hand, individuals with higher levels of physical activity may be prone to engage in risk-taking behaviours during flooding events, potentially leading to increased mortality rates [63]. These behaviours could involve actions such as entering floodwaters to cross a river or stream, safeguarding property and families (e.g. through activities like sandbagging homes and clearing drains), and participating in rescuing operations [63]. Surprisingly, current smokers demonstrated a decreased risk of flood-related mortality from all-cause deaths and neoplasms. We acknowledge that residual confounding, raising from unmeasured factors at follow-up, might contribute to these associations. Nevertheless, it is important to note that our study represents the first report of a higher mortality risk after long-term exposure to floods, highlighting the need for further investigations to validate this finding and explore potential underlying reasons.

Based on our research, flooding exposure is responsible for advancing a substantial number of deaths, with the impact persisting for up to 6 years. Our findings suggest that preventive interventions should be implemented peri- and post-flooding periods to reduce avoidable deaths due to flooding exposure. Following a flooding event, there is an increased risk of suicide within the first year. Therefore, timely provision of coping support and stress management is crucial to avert psychological illness, particularly among individuals in low socio-economic groups, those engaged in less physical activity, and non-moderate alcohol drinkers. In long-term rehabilitation, more resources should be allocated towards addressing the chronic medical conditions of populations that have been exposed to flooding, especially neurological well-being. It is also crucial to pay attention to the high-income population, although further research is needed to elucidate the underlying mechanisms behind their greater mortality risks associated with flooding exposure.

The limitations merit consideration. Our participants were residents in the UK who were more likely to live in less socioeconomically deprived areas, therefore, our results may not be generalizable to a whole population, especially people in low- and middle-income countries. Like most of cohort studies, covariates were collected at enrolment in the biobank. Due to the limited information on behavioural changes after the baseline examinations, we are unable to exclude the effect of behavioural changes on the risk estimates. However, most of the covariates (e.g. socio-economic status) were considered as effect modifiers rather than confounders, therefore, any changes in these factors should not have a substantial impact on our estimates. While the flood index accounts for cumulative exposure, it does not yet capture the potential differential impacts of distinct flood phases (warning, event, post-event) on mortality. Further research with a short-term design would be helpful to investigate the impacts of distinct flood phases. The destructive power of floods can differ based on factors like terrain, altitude, water management, drainage, urbanization, and building design. Therefore, a single severity label for an entire flood event may not fully capture the nuances of varying local experiences. Further research is needed to refine our exposure assessment as more detailed data becomes available. Lastly, it is likely for people to move after flooding exposure, with or without moving back into their homes. We assumed that participants did not move, which may have underestimated the effect of floods if an individual moved from an area with a high risk of flooding to an area with a lower risk of flooding. However, exposure to flooding can still have a long-term impact on them due to potential property damage and financial loss, even if people relocate to areas with a low risk of flooding in the aftermath of the event.

## Conclusions

In conclusion, this study provides robust epidemiological evidence for associations of long-term exposure to flooding with increased risk of mortality. The health consequences of flooding exposure can vary across different periods after the event. These findings contribute to a better understanding of the long-term impacts of flooding exposure and can help improve public health practices to reduce the disease burden associated with floods.

### Supplementary Information

**Additional file 1:** **Table S1.** Definitions of different severities of flood events. **Table S2.** The Akaike Information Criterion (AIC) and the Bayesian Information Criterion (BIC) of nonlinear and linear models. **Table S3.** Baseline characteristics of cases and matched controls enrolled in UK Biobank, including missing values. **Table S4.** Cumulative odds ratios of cause-specific mortality associated with per unit increase in flood index over lag years 0–5. **Table S5.** Cumulative odds ratios of all-cause mortality associated with per unit increase in monthly flood index over lag month 0–12. **Figure S1. **Nonlinear curves of the associations between flood index and all-cause and cause-specific mortality. **Figure S2.** Cumulative flood index of cases and controls during the six years before the date of death or the end of the follow-up. **Figure S3.** Cumulative odds ratios of all-cause and cause-specific mortality associated with per unit increase in flood index over lag years 0–5 using complete data after multiple imputation. **Figure S4.** Cumulative odds ratios of all-cause and cause-specific mortality associated with per unit increase in flood index over lag years 0–5 using different degrees of freedom for lag-response association of flood index. **Figure S5.** Cumulative odds ratios of all-cause and cause-specific mortality associated with per unit increase in flood index over lag years 0–5 using different degrees of freedom for mean temperature. **Figure S6.** Cumulative odds ratios of all-cause and cause-specific mortality associated with per unit increase in flood index over lag years 0–5 using different degrees of freedom for relative humidity. **Figure S7.** Cumulative odds ratios of all-cause and cause-specific mortality associated with per unit increase in flood index over lag years 0–5 after excluding deaths after 2020. **Figure S8.** Cumulative odds ratios of all-cause and cause-specific mortality associated with per unit increase in flood index over lag years 0–5 using different matching ratios.

## Data Availability

Data used in this study are available through registration on the UK Biobank.

## Abbreviations

AIC: Akaike Information Criterion
BIC: Bayesian Information Criterion
BMI: Body mass index
CI: Confidence interval
COVID-19: Coronavirus disease 2019
DFO: Dartmouth Flood Observatory
ERA-5: European Centre for Medium-Range Weather Forecasts Reanalysis v5
ICD-10: International Classification of Diseases, edition 10
IPAQ-SF: International Physical Activity Questionnaire-Short Form
MET-minutes/week: Metabolic equivalent minutes per week
OR: Odds ratio
PTSD: Post-traumatic stress disorder
SD: Standard deviation
TDI: Townsend deprivation index
