# Supplementary material for: Floods and cause-specific mortality in the UK: a nested case-control study
Source: BMC Med. 2024 May 7;22:188. doi: 10.1186/s12916-024-03412-0 (PMC11077877; doi:10.1186/s12916-024-03412-0)
Supplement: Supplementary file 1 — Additional file 1: Table S1. Definitions of different severities of flood events. Table S2. The Akaike Information Criterion (AIC) and the Bayesian Information Criterion (BIC) of nonlinear and linear models. Table S3. Baseline characteristics of cases and matched controls enrolled in UK Biobank, including missing values. Table S4. Cumulative odds ratios of cause-specific mortality associated with per unit increase in flood index over lag years 0–5. Table S5. Cumulative odds ratios of all-cause mortality associated with per unit increase in monthly flood index over lag month 0–12. Figure S1. Nonlinear curves of the associations between flood index and all-cause and cause-specific mortality. Figure S2. Cumulative flood index of cases and controls during the six years before the date of death or the end of the follow-up. Figure S3. Cumulative odds ratios of all-cause and cause-specific mortality associated with per unit increase in flood index over lag years 0–5 using complete data after multiple imputation. Figure S4. Cumulative odds ratios of all-cause and cause-specific mortality associated with per unit increase in flood index over lag years 0–5 using different degrees of freedom for lag-response association of flood index. Figure S5. Cumulative odds ratios of all-cause and cause-specific mortality associated with per unit increase in flood index over lag years 0–5 using different degrees of freedom for mean temperature. Figure S6. Cumulative odds ratios of all-cause and cause-specific mortality associated with per unit increase in flood index over lag years 0–5 using different degrees of freedom for relative humidity. Figure S7. Cumulative odds ratios of all-cause and cause-specific mortality associated with per unit increase in flood index over lag years 0–5 after excluding deaths after 2020. Figure S8. Cumulative odds ratios of all-cause and cause-specific mortality associated with per unit increase in flood index over lag years 0–5 using different matching rati [file 12916_2024_3412_MOESM1_ESM.docx]

**Floods and cause-specific mortality in the UK: a nested case-control study**

**Additional file 1**

**Table of Contents**

**Table S1**. Definitions of different severities of flood events.

**Table S2.** The Akaike Information Criterion (AIC) and the Bayesian Information Criterion (BIC) of nonlinear and linear models.

**Table S3.** Baseline characteristics of cases and matched controls enrolled in UK Biobank, including missing values.

**Table S4**. Cumulative odds ratios of cause-specific mortality associated with per unit increase in flood index over lag years 0–5.

**Table S5.** Cumulative odds ratios of all-cause mortality associated with per unit increase in monthly flood index over lag month 0–12.

**Figure S1.** Nonlinear curves of the associations between flood index and all-cause and cause-specific mortality.

**Figure S2.** Cumulative flood index of cases and controls during the six years before the date of death or the end of the follow-up.

**Figure S3**. Cumulative odds ratios of all-cause and cause-specific mortality associated with per unit increase in flood index over lag years 0–5 using complete data after multiple imputation.

**Figure S4**. Cumulative odds ratios of all-cause and cause-specific mortality associated with per unit increase in flood index over lag years 0–5 using different degrees of freedom for lag-response association of flood index.

**Figure S5**. Cumulative odds ratios of all-cause and cause-specific mortality associated with per unit increase in flood index over lag years 0–5 using different degrees of freedom for mean temperature.

**Figure S6**. Cumulative odds ratios of all-cause and cause-specific mortality associated with per unit increase in flood index over lag years 0–5 using different degrees of freedom for relative humidity.

**Figure S7**. Cumulative odds ratios of all-cause and cause-specific mortality associated with per unit increase in flood index over lag years 0–5 after excluding deaths after 2020.

**Figure S8**. Cumulative odds ratios of all-cause and cause-specific mortality associated with per unit increase in flood index over lag years 0–5 using different matching ratios.

**Table S1. Definitions of different severities of flood events.**

| **Severity class** | **Value** | **Definition*** |
| --- | --- | --- |
| No flood events | 0 | No flood events |
| Large flood events | 1 | Significant damage to structures or agriculture; fatalities; and/or 1–2 decades-long reported return period. |
| Very large events | 1.5 | With an estimated return period greater than 2 decades but less than 100 years, and/or with a local return period of 1–2 decades and affecting a large geographic region (> 5000 km^2^). |
| Extreme events | 2 | With an estimated return period greater than 100 years. |

***** The concept of a return period denotes the likelihood of natural disasters of equivalent intensity occurring. For instance, a flood event with a return period of 20 years indicates a 1/20 or 5% probability of transpiring within a single year. A greater return period correlates with increased severity concerning flood volume, inundation area, direct economic repercussions, and affected population.

**Table S2. The Akaike Information Criterion (AIC) and the Bayesian Information Criterion (BIC) of nonlinear and linear models.**

| Outcome | Linear analysis | |  | Nonlinear analysis | |
| --- | --- | --- | --- | --- | --- |
|  | AIC | BIC |  | AIC | BIC |
| All-cause | 591053.546 | 591087.166 |  | 612131.701 | 612140.106 |
| CVD | 101801.484 | 101828.756 |  | 105586.789 | 105593.607 |
| Respiratory | 31720.4761 | 31743.562 |  | 32720.9287 | 32726.7002 |
| Digestive | 14731.471 | 14752.0613 |  | 15455.2497 | 15460.3973 |
| Neurodegenerative | 28391.8473 | 28414.373 |  | 29069.537 | 29075.1684 |
| Mental | 7843.39484 | 7861.41199 |  | 8037.17828 | 8041.68257 |
| Neoplasms | 287459.782 | 287490.797 |  | 298296.824 | 298304.578 |
| Suicide | 2575.84303 | 2591.01508 |  | 2864.14803 | 2867.94104 |

Abbreviation: AIC, Akaike Information Criterion; BIC, Bayesian Information Criterion.

**Table S3. Baseline characteristics of cases and matched controls enrolled in UK Biobank, including missing values.**

|  | **Overall** | **Case** | **Control** |
| --- | --- | --- | --- |
| N* | 291,414 | 33,021 | 258,393 |
| Age, mean (SD) | 61.32 (6.44) | 61.62 (6.45) | 61.28 (6.44) |
| Male (%) | 170549 (58.5) | 19591 (59.3) | 150958 (58.4) |
| White ethnicity (%） | 281175 (96.5) | 31867 (96.5) | 249308 (96.5) |
| BMI, mean (SD)† | 27.64 (4.61) | 28.28 (5.41) | 27.56 (4.49) |
| Education attainment (%) |  |  |  |
| Low | 202677 (69.5) | 24606 (74.5) | 178071 (68.9) |
| High | 83107 (28.5) | 7528 (22.8) | 75579 (29.2) |
| Missing values | 5630 (1.9) | 887 (2.7) | 4743 (1.8) |
| Household income (%) |  |  |  |
| < 31,000 | 141030 (48.4) | 18515 (56.1) | 122515 (47.4) |
| ≥31,000 | 101733 (34.9) | 8200 (24.8) | 93533 (36.2) |
| Missing values | 48651 (16.7) | 6306 (19.1) | 42345 (16.4) |
| Physical activity (%) |  |  |  |
| Low | 42663 (14.6) | 6104 (18.5) | 36559 (14.1) |
| Middle | 57267 (19.7) | 6223 (18.8) | 51044 (19.8) |
| High | 132489 (45.5) | 12956 (39.2) | 119533 (46.3) |
| Missing values | 58995 (20.2) | 7738 (23.4) | 51257 (19.8) |
| Smoking (%) |  |  |  |
| Never | 144356 (49.5) | 12588 (38.1) | 131768 (51.0) |
| Previous | 116627 (40.0) | 13790 (41.8) | 102837 (39.8) |
| Current | 29076 (10.0) | 6417 (19.4) | 22659 (8.8) |
| Missing values | 1355 (0.5) | 226 (0.7) | 1129 (0.4) |
| Alcohol intake |  |  |  |
| Moderate | 53060 (18.2) | 4926 (14.9) | 48134 (18.6) |
| Non-moderate | 164134 (56.3) | 17408 (52.7) | 146726 (56.8) |
| Missing values | 74220 (25.5) | 10687 (32.4) | 63533 (24.6) |
| Suboptimal diet |  |  |  |
| Yes | 122724 (42.1) | 15160 (45.9) | 107564 (41.6) |
| No | 158935 (54.5) | 16257 (49.2) | 142678 (55.2) |
| Missing values | 9755 (3.3) | 1604 (4.9) | 8151 (3.2) |
| Deprivation |  |  |  |
| Low | 151300 (51.9) | 14462 (43.8) | 136838 (53.0) |
| High | 139808 (48.0) | 18529 (56.1) | 121279 (46.9) |
| Missing values | 306 (0.1) | 30 (0.1) | 276 (0.1) |
| Health rating |  |  |  |
| Poor | 14296 (4.9) | 4383 (13.3) | 9913 (3.8) |
| Fair | 64233 (22.0) | 10066 (30.5) | 54167 (21.0) |
| Good | 167285 (57.4) | 15241 (46.2) | 152044 (58.8) |
| Excellent | 44269 (15.2) | 3025 (9.2) | 41244 (16.0) |
| Missing values | 1331 (0.5) | 306 (0.9) | 1025 (0.4) |

*12 case-control sets are incomplete.

†There are 1232 missing values for controls and 449 missing values for cases.

**Table S4. Cumulative odds ratios of cause-specific mortality associated with per unit increase in flood index over lag years 0–5.**

| **Cause of death** | **Number of cases** | **Odds ratio (95% CI)** | | |
| --- | --- | --- | --- | --- |
|  |  | Model 1* | Model 2† | Model 3‡ |
| All-cause | 33021 | 1.092 (1.090 to 1.093) | 1.090 (1.088 to 1.091) | 1.067 (1.063 to 1.071) |
| CVD | 6754 | 1.084 (1.081 to 1.087) | 1.080 (1.077 to 1.084) | 1.051 (1.042 to 1.059) |
| Respiratory | 2372 | 1.086 (1.081 to 1.091) | 1.081 (1.075 to 1.087) | 1.062 (1.045 to 1.080) |
| Digestive | 1271 | 1.079 (1.072 to 1.086) | 1.071 (1.063 to 1.079) | 1.031 (1.011 to 1.052) |
| Neurodegenerative | 2062 | 1.064 (1.058 to 1.071) | 1.062 (1.055 to 1.069) | 1.068 (1.050 to 1.087) |
| Mental | 668 | 1.066 (1.055 to 1.078) | 1.055 (1.041 to 1.070) | 1.047 (1.008 to 1.087) |
| Neoplasms | 17220 | 1.087 (1.085 to 1.089) | 1.087 (1.085 to 1.089) | 1.063 (1.058 to 1.068) |
| Suicide | 328 | 1.103 (1.090 to 1.116) | 1.101 (1.087 to 1.115) | 1.052 (1.018 to 1.088) |

* Model 1 was crude model;

† Model 2 was adjusted for matching factors and socio-economic factors (education, household income, and deprivation);

‡ Model 3 was adjusted for matching factors, socio-economic factors, BMI, physical activity, smoking, alcohol consumption, diet score, overall health rating, and assessment center.

**Table S5.** **Cumulative odds ratios of all-cause mortality associated with per unit increase in monthly flood index over lag month 0–12.**

| **Lag structure (month)** | **Odds Ratio (95% CI)** |
| --- | --- |
| 0 | 1.005 (1.005 to 1.006) |
| 1 | 1.006 (1.006 to 1.007) |
| 2 | 1.007 (1.007 to 1.008) |
| 3 | 1.008 (1.008 to 1.009) |
| 4 | 1.009 (1.009 to 1.010) |
| 5 | 1.011 (1.010 to 1.011) |
| 6 | 1.012 (1.011 to 1.012) |
| 7 | 1.013 (1.012 to 1.013) |
| 8 | 1.014 (1.013 to 1.014) |
| 9 | 1.015 (1.014 to 1.015) |
| 10 | 1.016 (1.015 to 1.016) |
| 11 | 1.017 (1.016 to 1.017) |
| 12 | 1.018 (1.017 to 1.018) |


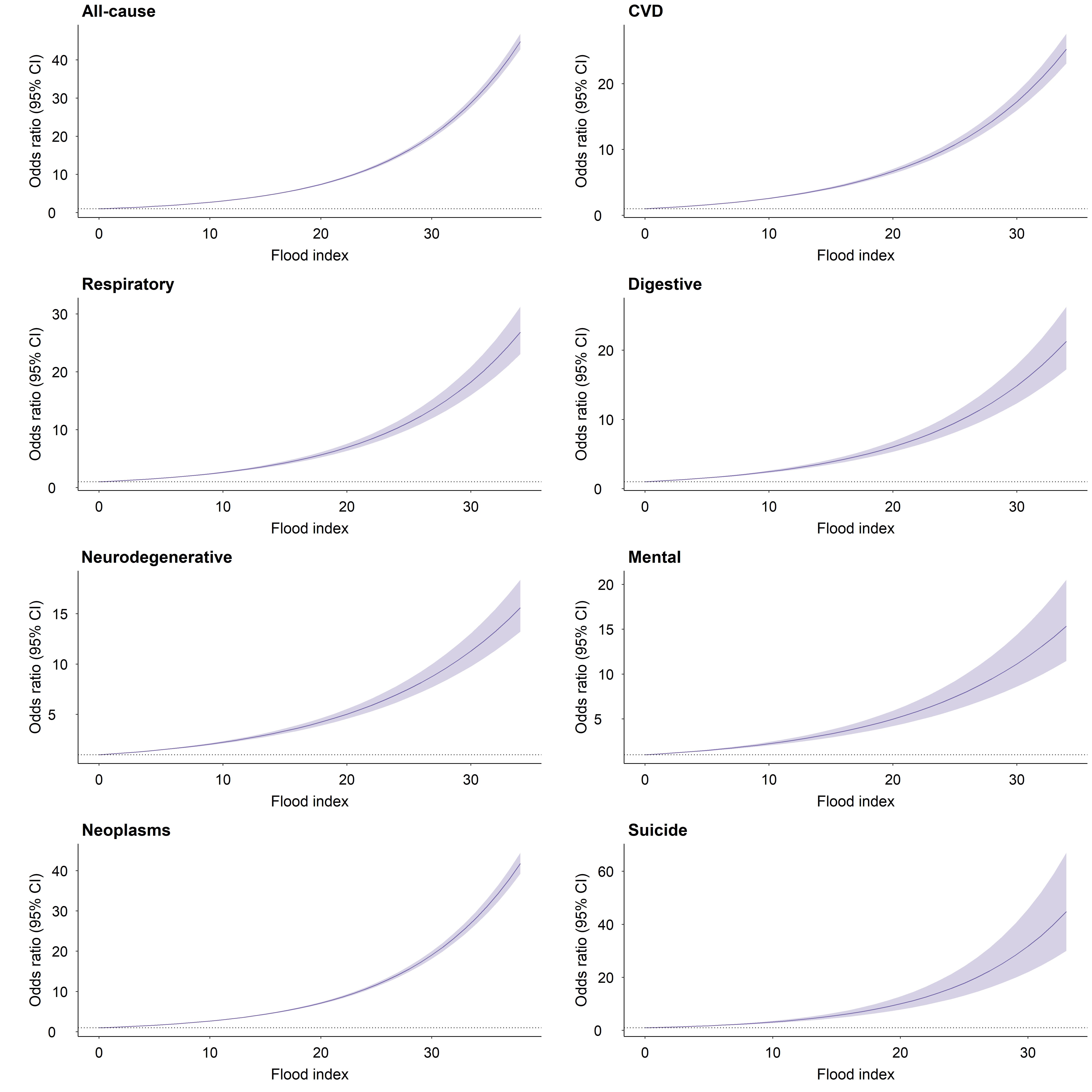


**Figure S1. Nonlinear curves of the associations between flood index and all-cause and cause-specific mortality.**


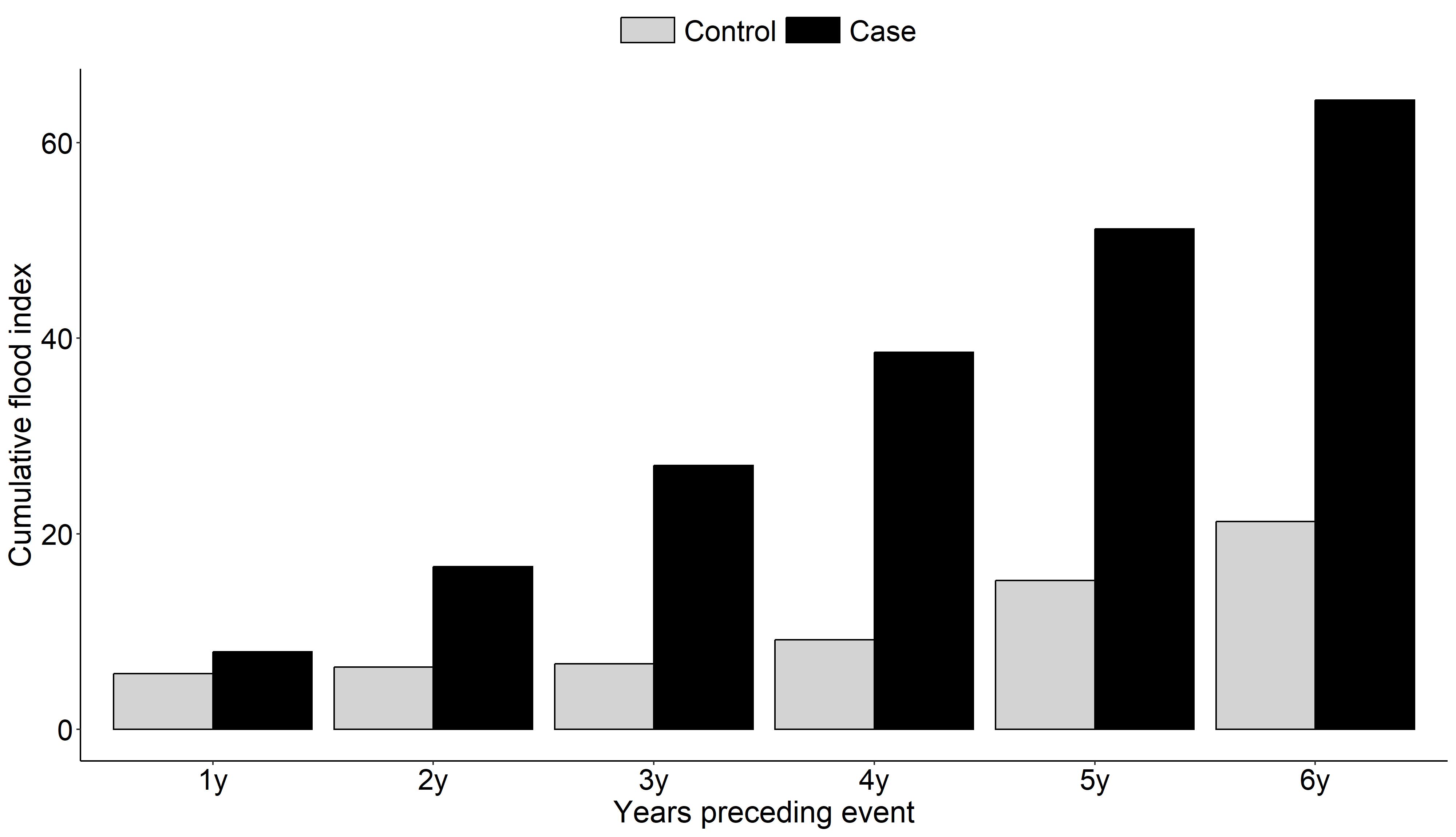


**Figure S2. Cumulative flood index of cases and controls during the six years before the date of death or the end of the follow-up.**


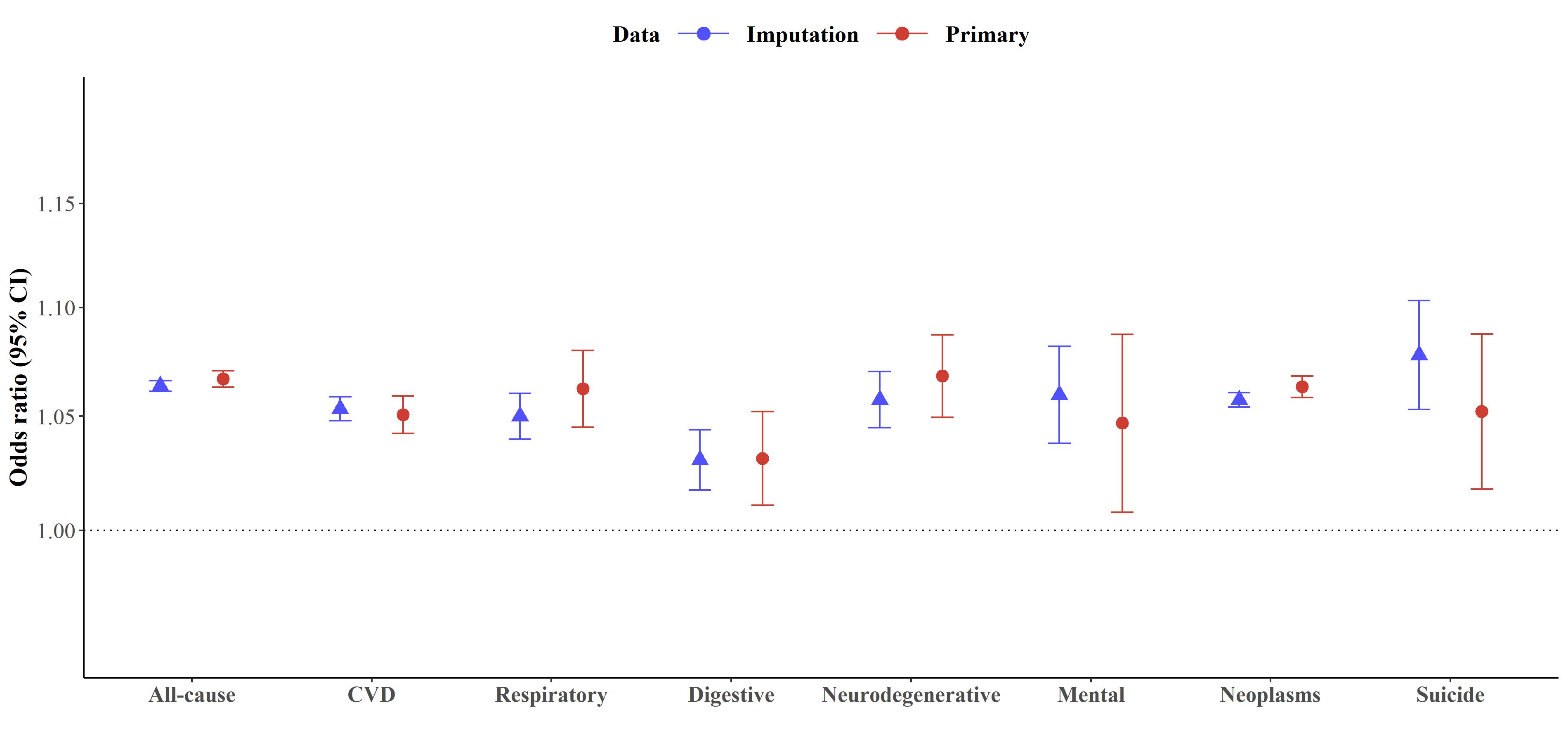


**Figure S3. Cumulative odds ratios of all-cause and cause-specific mortality associated with per unit increase in flood index over lag years 0–5 using complete data after multiple imputation.**


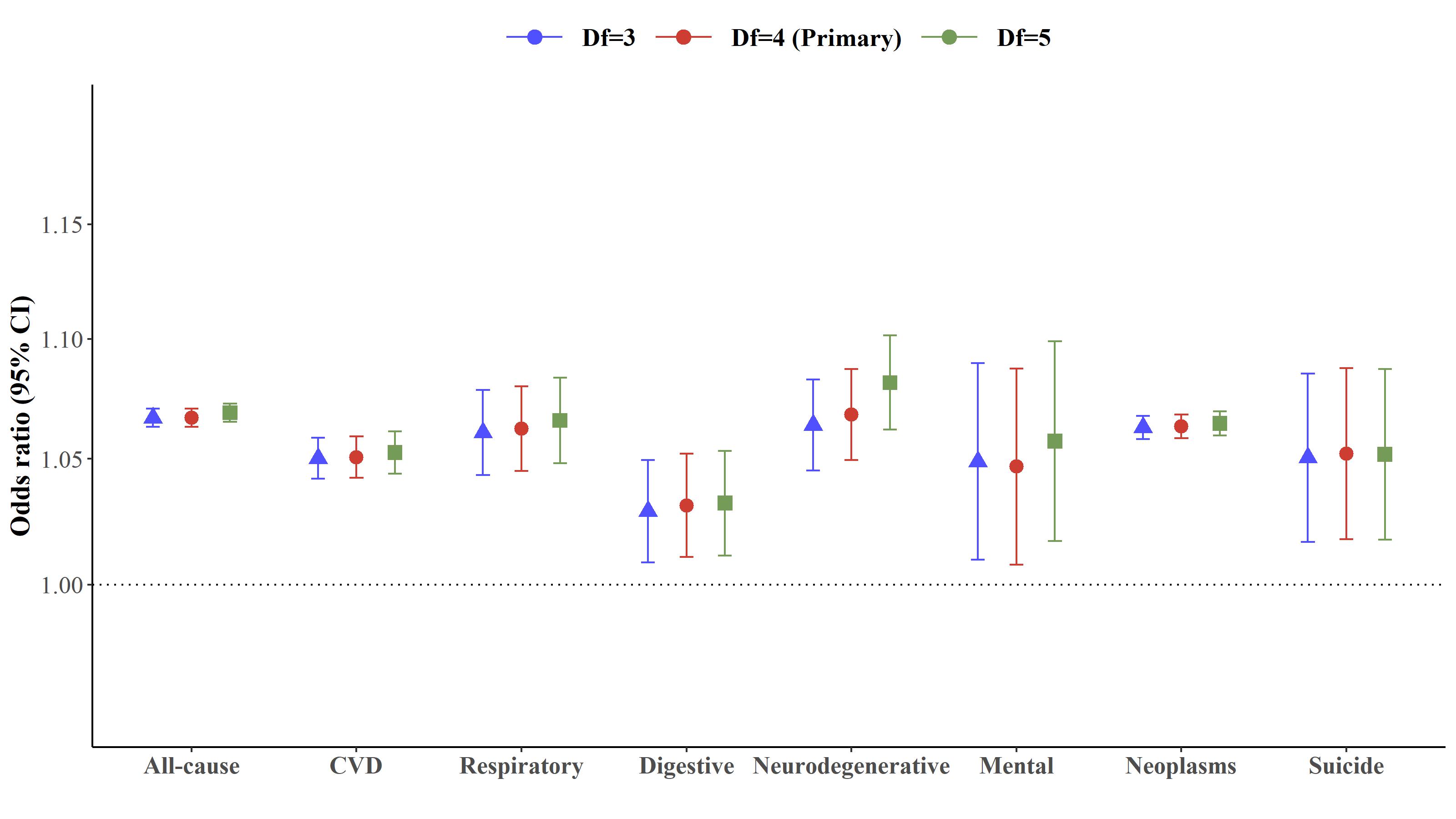


**Figure S4. Cumulative odds ratios of all-cause and cause-specific mortality associated with per unit increase in flood index over lag years 0–5 using different degrees of freedom for lag-response association of flood index.**


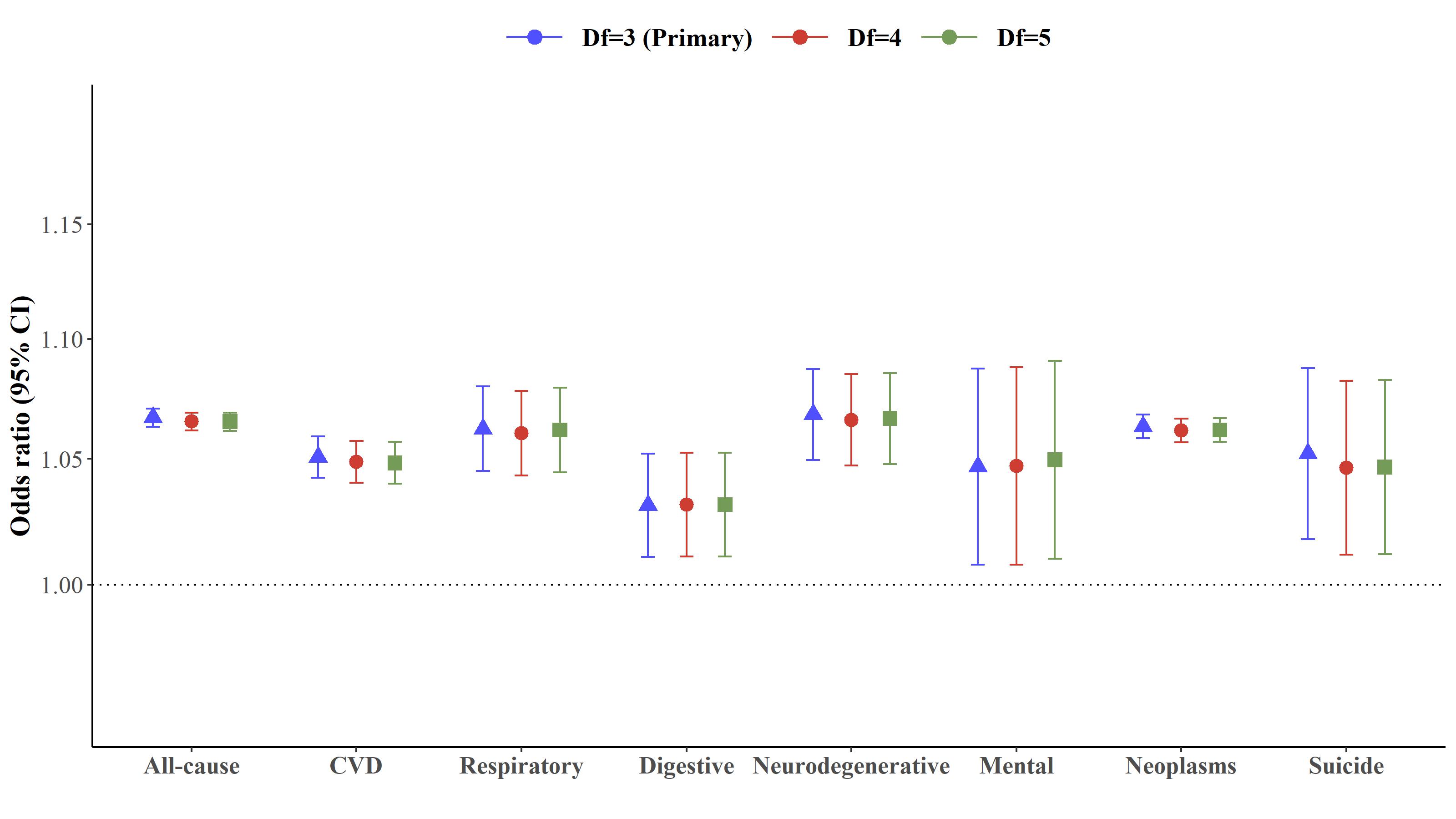


**Figure S5. Cumulative odds ratios of all-cause and cause-specific mortality associated with per unit increase in flood index over lag years 0–5 using different degrees of freedom for mean temperature.**


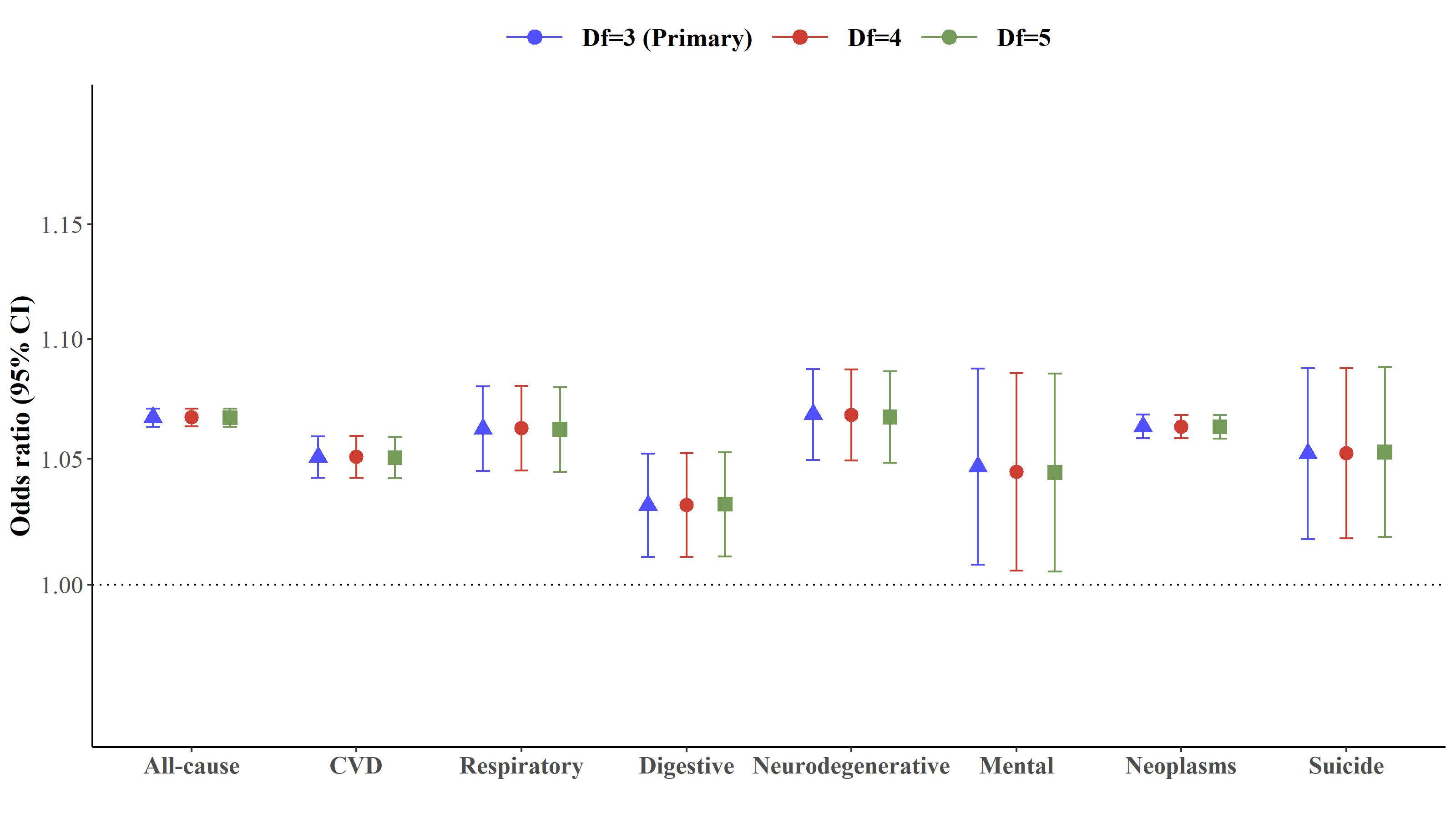


**Figure S6. Cumulative odds ratios of all-cause and cause-specific mortality associated with per unit increase in flood index over lag years 0–5 using different degrees of freedom for relative humidity.**

**
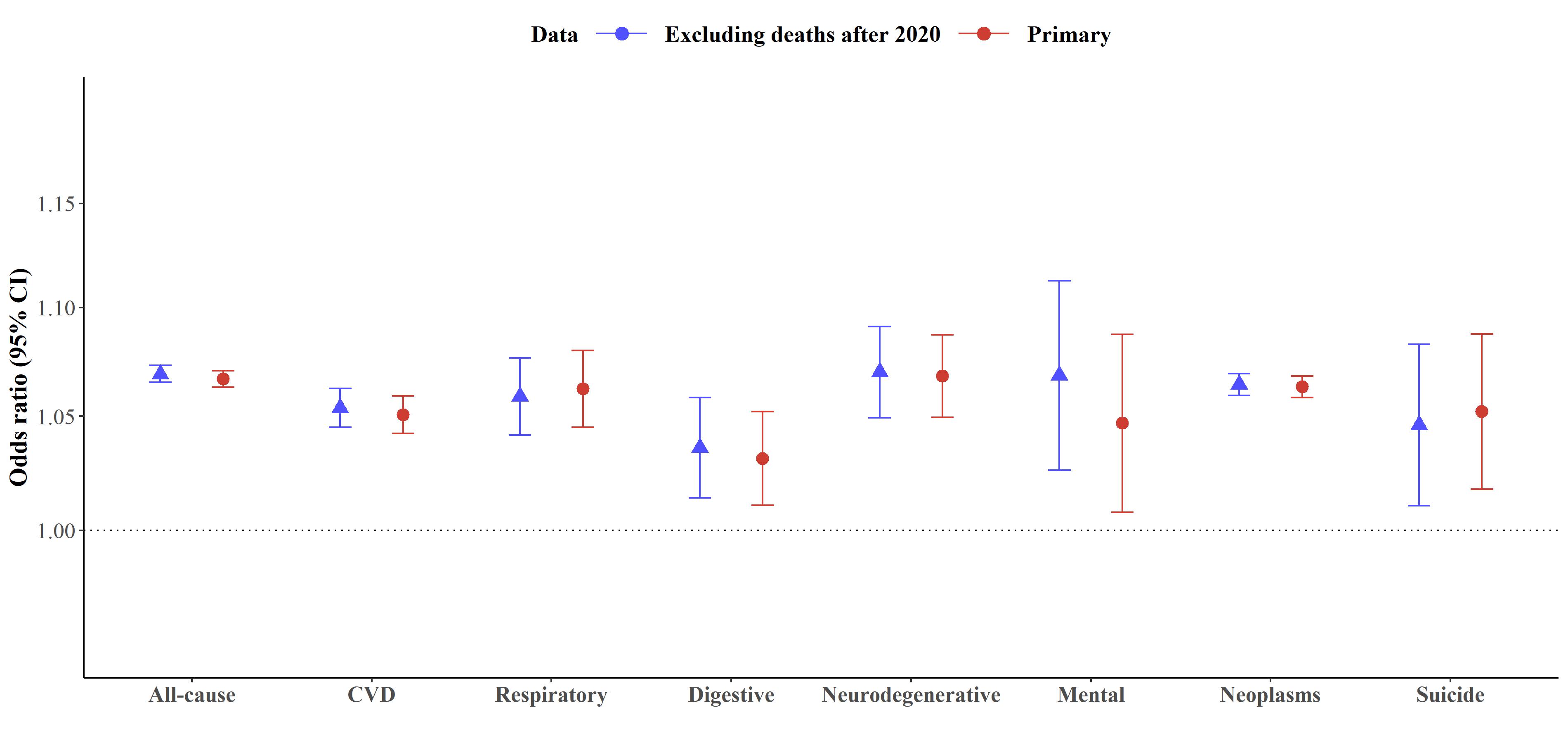
Figure S7. Cumulative odds ratio of all-cause and cause-specific mortality associated with per unit increase in flood index over lag years 0–5 after excluding deaths after 2020.**


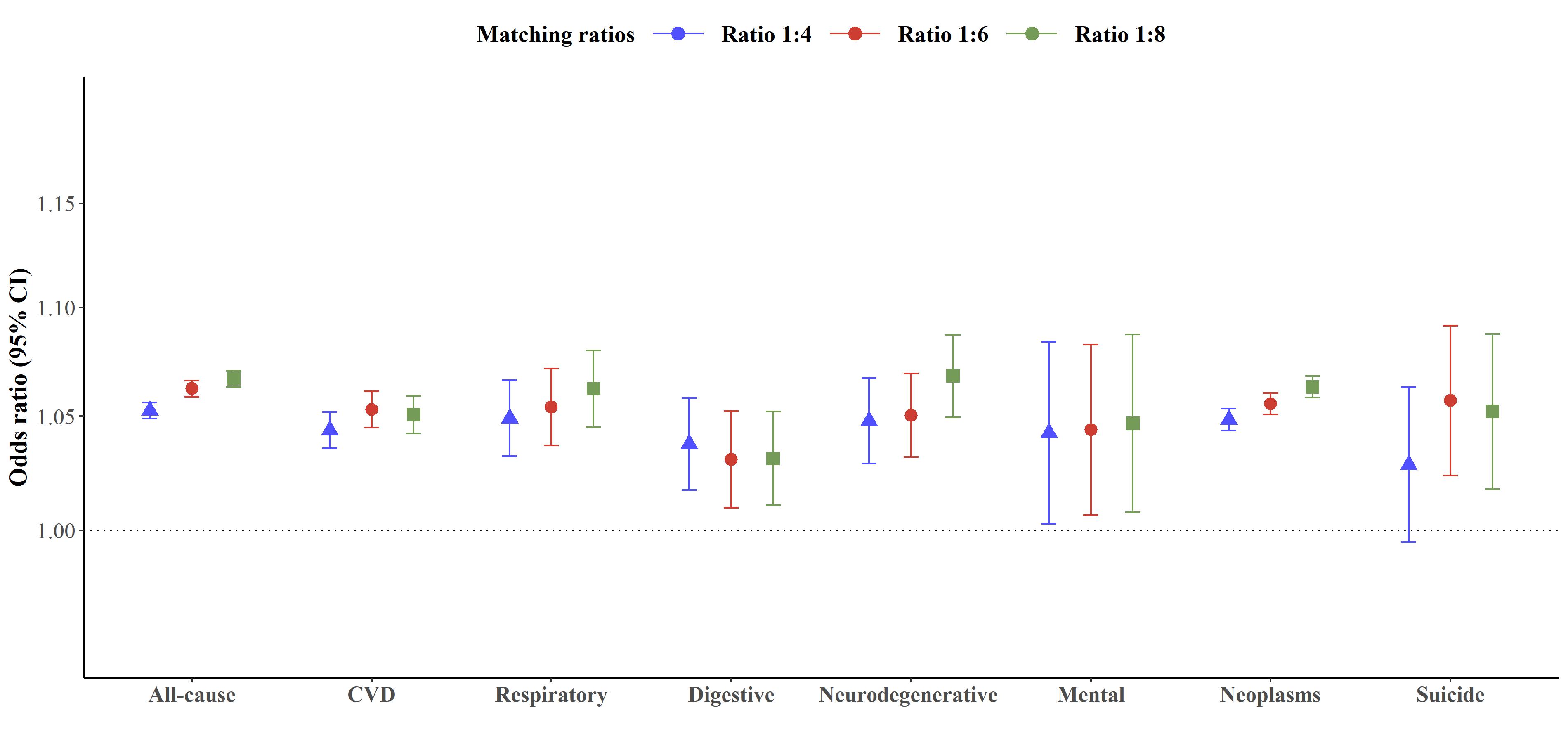


**Figure S8. Cumulative odds ratios of all-cause and cause-specific mortality associated with per unit increase in flood index over lag years 0–5 using different matching ratios.**
